# High-Temperature Energy Storage Performance of Polyimide Nanocomposites Enhanced by Core–Shell BT-BMT@SiO_2_

**DOI:** 10.3390/polym18141784

**Published:** 2026-07-21

**Authors:** Zunpeng Feng, Xingyu Hou, Sitian Ren, Xinyao Zhuang, Wei Chen, Haoran Liu, Chaoqiong Zhu, Ziming Cai, Peizhong Feng

**Affiliations:** 1 School of Materials Science and Physics, China University of Mining and Technology, Xuzhou 221116, China; fengzunpeng@cumt.edu.cn (Z.F.); hxy_980522@163.com (X.H.); ren041346@163.com (S.R.); 14240159@cumt.edu.cn (X.Z.); 18895795431@163.com (W.C.); ts25180114p31@cumt.edu.cn (H.L.); zhucq@cumt.edu.cn (C.Z.); 2 Jiangsu Key Laboratory for Clean Utilization of Carbon Resources, Xuzhou 221116, China

**Keywords:** high-temperature energy storage, core–shell structure, dielectric, relaxor ferroelectric, polyimide, nanocomposites

## Abstract

Advanced electrical and electronic systems are placing increasingly stringent demands on high-temperature dielectric capacitors. Polyimide (PI) possesses excellent thermal stability, but its low dielectric constant and breakdown strength limit its energy storage performance. To address this, a nanocomposite was designed consisting of the amorphous SiO_2_-coated relaxor ferroelectric ceramic 0.6BaTiO_3_-0.4Bi(Mg_0.5_Ti_0.5_)O_3_ (BT-BMT@SiO_2_) and PI. This core–shell structure improves the organic-inorganic interface and mitigates dielectric mismatch, thereby synergistically enhancing the composite’s dielectric constant, breakdown strength, and energy storage performance. At 150 °C, the breakdown field strength and maximum discharge energy density of the 0.25 vol.% BT-BMT@SiO_2_/PI composite reached 381.81 MV/m and 1.14 J/cm^3^, respectively, representing increases of 32% and 67% compared to pure PI. These results indicate that the synergistic design of relaxor ferroelectric ceramics and core–shell interfaces is an effective strategy for enhancing the high-temperature energy storage performance of PI-based dielectric materials, providing important guidance for the development of high-performance capacitor materials.

## 1. Introduction

Electrostatic capacitors are widely used in microelectronic integrated circuits, new energy vehicles, voltage-source converter-based high-voltage direct current (HVDC) systems, and the aerospace industry, owing to their high power density, fast charge–discharge rates, excellent cycling stability, high operating voltage, and superior ripple current handling capability [1,2,3,4]. The dielectric constant (*ε*_r_) and breakdown strength (*E*_b_) are the key parameters governing energy storage performance [5,6,7]. Ceramic capacitors deliver high polarization and outstanding stability, making them particularly suited for high-temperature, high-frequency energy storage, yet they are limited by relatively low breakdown strength [8,9,10]. In contrast, film capacitors based on polymer dielectrics have drawn increasing attention for their extremely high *E*_b_ and good flexibility [11,12,13]. Biaxially oriented polypropylene (BOPP) has become the most successful commercial metallized film capacitor material thanks to its excellent dielectric strength, superior self-healing properties, and low manufacturing cost [2,14]. However, with the rapid adoption of wide-bandgap semiconductors such as SiC and GaN in power electronics, the low dielectric constant (*ε*_r_~2.2) and limited long-term operating temperature (≤105 °C) of BOPP films make it difficult to meet the demands of extreme environments such as electric vehicles, electromagnetic catapult systems, and underground oil and gas exploration [15,16]. High-*T*_g_ polymers including polyether ether ketone (PEEK, *T*_g_~150 °C), polyimide (PI, *T*_g_~360 °C), polycarbonate (PC, *T*_g_~150 °C), and polyetherimide (PEI, *T*_g_~217 °C) contain a large number of highly conjugated aromatic ring that provide excellent mechanical strength and thermal stability at elevated temperatures [1,17,18]. Nevertheless, these polymers typically lack strongly polar functional groups and remain constrained by inherently low dielectric constants [19,20].

Incorporating high-*ε*_r_ ceramic fillers is one of the most direct and effective strategies for increasing the dielectric constant of polymers [17]. Inorganic nanofillers with high relative permittivity (*ε*_r_), such as barium titanate (BT), barium strontium titanate (BST), and barium zirconium calcium titanate (BZCT), can markedly enhance polarization in composite dielectric films even at low loadings [21,22,23]. However, their inherently high dielectric loss (tan δ) significantly impairs charge–discharge efficiency [24]. Compared with conventional ferroelectrics like BT and BST, relaxor ferroelectrics have recently emerged as highly attractive candidates for dielectric energy storage because their nanoscale polar domains enable rapid polarization switching, resulting in slim polarization-electric field (*P*-*E*) loops with high maximum polarization, low remanent polarization, and thus simultaneously high energy density and efficiency [25,26]. Introducing relaxor ferroelectric ceramic particles into high-temperature-resistant polymers is expected to alleviate the low dielectric constant and energy efficiency of high-temperature composite dielectrics, thereby improving energy storage density. For instance, Guo et al. prepared BNKT-BST relaxor ferroelectric ceramics with high dielectric constant and charge–discharge efficiency, and their incorporation into PEI significantly enhanced the dielectric constant, breakdown strength, and mechanical properties of the composite [27]. The energy storage performance of the resulting composite was improved by 171% compared to pure PEI [27].

Among various relaxor compositions, the solid solution (1 − x)BaTiO_3_-xBi(Mg_0.5_Ti_0.5_)O_3_ has drawn particular attention for its superior energy storage performance near the morphotropic phase boundary [28,29,30,31,32]. The incorporation of Bi(Mg_0.5_Ti_0.5_)O_3_ into the BaTiO_3_ lattice disrupts long-range ferroelectric order by introducing chemical and charge inhomogeneity, which promotes the formation of highly dynamic polar nanoregions and significantly suppresses hysteresis loss while retaining a large dielectric response [33,34,35]. The composition 0.6BaTiO_3_-0.4Bi(Mg_0.5_Ti_0.5_)O_3_ (BT-BMT) combines a high maximum electric displacement, a low residual displacement, and a moderate dielectric constant, thereby exhibiting the best overall performance [36,37]. The BT-BMT nanoparticles were synthesized following a previously reported solid-state route, ensuring the reliability and reproducibility of the core material [36].

Nevertheless, the large *ε*_r_ mismatch between filler and matrix still leads to charge accumulation and electric field distortion at the interface, triggering premature breakdown and limiting energy storage density (*U*_e_) [24,38]. Moreover, the physical property mismatch between the polymer and the ceramic fillers further causes interfacial incompatibility, compromising the compactness and structural uniformity of the composite and increasing the risk of premature dielectric failure [39,40]. Studies show that surface functionalization of inorganic fillers with organic or inorganic coatings can effectively mitigate interfacial dielectric mismatch and improve compatibility [41,42,43]. Inorganic coatings are preferred over organic ones because they avoid the additional losses caused by molecular relaxation and the reduced thermal stability of organic layers at high temperatures [44,45,46]. Therefore, engineering the filler-polymer interface and forming a suitable inorganic shell on the particle surface are key to synergistically enhancing both *ε*_r_ and *E*_b_. Among inorganic coating materials, SiO_2_ has attracted considerable attention owing to its moderate dielectric constant (~4) and excellent electrical insulation properties [47,48]. For instance, oriented core–shell BaTiO_3_@SiO_2_ nanofibers embedded in a PEI matrix were demonstrated to simultaneously deliver high dielectric constant, high breakdown strength, and outstanding energy efficiency [47]. Similarly, ultrafine core–shell BaTiO_3_@SiO_2_ structures have been shown to effectively enhance the energy density of nanocomposite capacitors [48]. Moreover, a sandwich-structured poly(vinylidene fluoride) (PVDF)-based composite filled with SiO_2_-coated BT-BMT nanofibers has demonstrated significantly improved room-temperature energy storage density; however, to the best of our knowledge, its potential for high-temperature energy storage composite has not yet been explored [49].

In this work, core–shell-structured 0.6BaTiO_3_-0.4Bi(Mg_0.5_Ti_0.5_)O_3_@SiO_2_ (BT-BMT@SiO_2_) relaxor ferroelectric nanoparticles were designed and synthesized, and subsequently incorporated into a polyimide (PI) matrix to regulate the interfacial matching and dielectric compatibility between the inorganic filler and the polymer matrix, thereby improving the high-temperature energy storage performance of the nanocomposite. The BT-BMT core delivers high maximum electric displacement (*D*_max_), low residual electric displacement (*D*_r_), and a narrow electric displacement-electric field (*D*-*E*) loop. The SiO_2_ shell, rich in surface hydroxyl groups, improves interface with PI, while its moderate dielectric constant (~4) effectively alleviates the dielectric mismatch between the low-*ε*_r_ PI matrix (~3.2) and the high-*ε*_r_ BT-BMT fillers (>1000) [19,36,47,48]. This suppresses local electric-field distortion and improves breakdown strength (*E*_b_). By combining the favorable organic-inorganic interface and dielectric compatibility of SiO_2_ with the unique advantages of BT-BMT (high *D*_max_, low *D*_r_), the BT-BMT@SiO_2_/PI nanocomposite achieves simultaneous enhancement in dielectric constant, breakdown field strength, energy efficiency, and energy storage density.

## 2. Materials and Methods

### 2.1. Materials

Pyromellitic dianhydride (PMDA, ≥99%) and 4,4′-oxydianiline (ODA, 98%) were purchased from Shanghai Aladdin Biochemical Technology Co., Ltd. (Shanghai, China). N, N′-dimethyl-acetamide (DMAc, containing molecular sieves, 99.8%), titanium dioxide (TiO_2_, 99.99%), bismuth trioxide (Bi_2_O_3_, 99.99%), barium carbonate (Ba_2_CO_3_, 99.99%), acetic acid (≥99.5%), ammonia water (≥99%), tetraethyl orthosilicate (≥99%), and anhydrous ethanol (≥99.8%) were purchased from Shanghai Hushi Laboratory Equipment Co., Ltd. (Shanghai, China). Magnesium oxide (MgO, ≥99.99%) was purchased from Sinopharm Chemical Reagents Co., Ltd. (Shanghai, China). Prior to use, PMDA and ODA were dried in a vacuum oven at 60 °C for 10 h to remove absorbed moisture, thereby preventing the hydrolysis of the dianhydride and ensuring the precise stoichiometric balance required for high-molecular-weight polyimide synthesis.

### 2.2. BT-BMT Nanoparticles and Their Ceramic Preparation

Synthesis of 0.6BaTiO_3_-0.4Bi(Mg_0.5_Ti_0.5_)O_3_ (BT-BMT) nanoparticles and fabrication of ceramic discs: The BT-BMT nanoparticles were synthesized following a previously reported method [36]. Stoichiometric amounts of Bi_2_O_3_, TiO_2_, MgO, and Ba_2_CO_3_ were first ball-milled at 300 rpm for 4 h. The resulting slurry was dried at 80 °C for 8 h to obtain the precursor powder, which was then pre-sintered at 900 °C, ball-milled again at 400 rpm for 12 h, and finally dried to yield BT-BMT nanoparticles. To prepare ceramic discs for electrical testing, the as-synthesized nanoparticles were mixed with a polyvinyl alcohol (PVA) solution, pressed at 200 MPa, degassed, and sintered in a muffle furnace. The sintered ceramics were mechanically ground and polished, and silver electrodes were deposited onto their surfaces via magnetron sputtering.

Preparation of BT-BMT@SiO_2_ nanoparticles: First, a specific amount of BT-BMT nanoparticles was weighed. A pre-prepared solution of ethyl orthosilicate was added based on a SiO_2_ coating ratio of 0.4 wt.%. The mixture was stirred for 1 h. Ammonia water was then slowly added dropwise while stirring to adjust the solution’s pH. Stirring continued for another hour, and the resulting BT-BMT@SiO_2_ nanoparticles were finally dried.

### 2.3. Preparation of Polyimide Film and Its Nanocomposites

Preparation of polyimide film: A specific amount of ODA was placed in a three-neck flask under a nitrogen atmosphere, and N, N-dimethylacetamide (DMAc) solvent was added. The mixture was stirred at room temperature for 2 h until completely dissolved. Subsequently, PMDA was added in four batches to the above solution at a molar ratio of PMDA to ODA of 1.03:1, and the mixture was stirred for 12 h to obtain a polyamic acid (PAA) precursor solution. The PAA solution was then poured onto a clean glass plate and spread into a film, which was treated under vacuum at 60 °C for 5 h to remove bubbles. Thermal imidization was then performed: holding at 80 °C for 30 min, 100 °C for 1 h, 200 °C for 1 h, and 300 °C and 350 °C for 1 h each, with a heating rate of 5 °C·min^−1^ for all stages. Finally, the film was peeled off to yield a pure polyimide film approximately 15 μm thick.

Preparation of nanocomposites: The nanocomposite films were fabricated via a solution mixing method. A specific mass of inorganic nanoparticles was first ultrasonically dispersed in DMAc for 1 h to obtain a homogeneous suspension. This suspension was then added to a PAA solution and stirred continuously for 12 h. The subsequent steps for film casting and thermal imidization followed the procedure established for the preparation of pure polyimide films.

### 2.4. Characterization

The microstructure of BT-BMT@SiO_2_ was observed using a transmission electron microscope (TEM, Thermo Fisher, Waltham, MA, USA, Tecnai G2 F20) operated at an accelerating voltage of 200 kV. The morphology of the BT-BMT inorganic nanoparticles and the cross-sectional microstructure of the nanocomposite films were examined using a field-emission scanning electron microscope (FE-SEM, Hitachi, Tokyo, Japan, SU8220) at an accelerating voltage of 10 kV. The cross-sections of the films were prepared by cryo-fracturing in liquid nitrogen. Fourier-transform infrared (FT-IR) spectra of the inorganic nanoparticles and composites were measured using a spectrometer (Bruker, Billerica, MA, USA, TENSOR II) in attenuated total reflectance (ATR) mode over the wavelength range of 2000 cm^−1^ to 450 cm^−1^. The crystalline phase structures of the inorganic nanoparticles and composites were characterized by X-ray diffractometer (XRD, Bruker, D8-Advance) using Cu Kα radiation (λ = 1.5406 Å). The diffractometer was operated at 40 kV and 40 mA, and patterns were collected in the 2*θ* range of 20–80° with a step size of 0.02° and a scan speed of 10°/min. The thermal stability of the composite films was evaluated by thermogravimetric analysis (TGA) from room temperature to 900 °C, and their thermal transition behavior was characterized by differential scanning calorimetry (DSC) over the range of 100 °C to 350 °C. Both measurements were performed using a simultaneous thermal analyzer (NETZSCH, Selb, Germany, STA449F5) at a heating rate of 10 °C/min under a nitrogen atmosphere. Mechanical properties of the composites were measured with a digital universal testing machine (ZhiQu, Dongguan, China, ZQ990B). Film specimens were cut into standard dumbbell-shaped specimens (narrow width 4 mm, gauge length 20 mm) and tested at a speed of 100 mm/min. At least five specimens were measured for each sample to obtain average values. Prior to dielectric and ferroelectric testing, circular gold/silver electrodes (3 mm in diameter) were deposited on the surfaces of ceramic substrates or nanocomposites via magnetron sputtering. The dielectric properties of the composite materials were measured using a dielectric testing system (LCR, Wayne Kerr, Bognor Regis, UK, WK6500B) at temperatures of 25 °C and 150 °C and frequencies ranging from 10^2^ Hz to 10^6^ Hz. A ferroelectric analyzer (PolyK Technologies, State College, PA, USA, PK-CPE1801) was used to test the hysteresis loops, breakdown field strength, and energy storage density of ceramic plates and polymer films at 25 °C and 150 °C. The testing environment was silicone oil, and the testing frequency for the hysteresis loops was 10 Hz.

## 3. Results and Discussion

**Figure 1:**
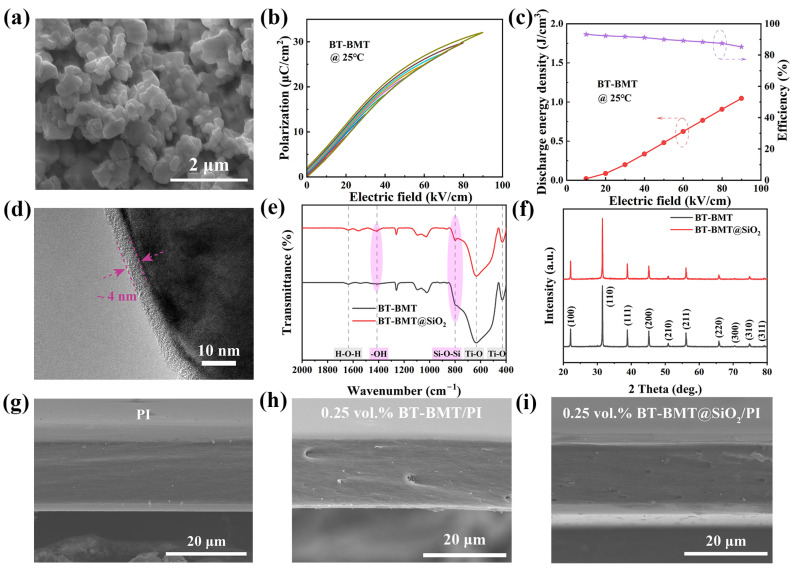
(**a**) SEM morphology of BT-BMT nanoparticles, (**b**) hysteresis loop and (**c**) energy storage performance of BT-BMT ceramic plates at 25 °C under different applied electric fields, (**d**) HRTEM image of BT-BMT@SiO_2_ particles, (**e**) FT-IR spectra and (**f**) XRD patterns of BT-BMT and BT-BMT@SiO_2_ ceramic particles, (**g**–**i**) cross-sectional SEM images of pure PI, 0.25 vol.% BT-BMT/PI and 0.25 vol.% BT-BMT@SiO_2_/PI composites.

Figure 1a shows the SEM morphology of BT-BMT ceramic particles after pre-firing at 900 °C and secondary ball milling. The particles are generally dense and free of obvious defects. The particle size is concentrated between 200 and 300 nm. Hysteresis loop testing (Figure 1b) indicates that the material exhibits typical relaxor ferroelectric characteristics: a narrow hysteresis loop, low coercive field, no saturation plateau, and low residual polarization. The polarization intensity increases continuously with the electric field, reaching 32 μC/cm^2^ at 90 kV/cm, demonstrating excellent polarization capacity and making it suitable as an inorganic filler for polymer matrices. Evaluation of energy storage performance (Figure 1c) shows that the discharge energy density increases with the electric field, reaching up to 1.04 J/cm^3^ (90 kV/cm). Although the efficiency decreases slightly, it remains above 80%, demonstrating the advantages of relaxor ferroelectrics in energy storage applications.

The TEM image (Figure 1d) clearly reveals the core–shell morphology of BT-BMT@SiO_2_: the surface of the BT-BMT grains is coated with a uniform amorphous SiO_2_ layer approximately 4 nm thick. FT-IR and XRD further confirm this core–shell structure and its chemical stability. In the FT-IR spectrum (Figure 1e), the Ti-O vibrational peaks at 635 cm^−1^ and 430 cm^−1^ confirm the formation of a perovskite metal–oxygen framework. The absorption peaks at 1639 cm^−1^ and 1414 cm^−1^ correspond to the H-O-H bending vibration and the -OH stretching vibration of adsorbed water molecules, respectively, with the -OH peak being enhanced after coating, which is attributed to the introduction of additional hydroxyl groups by the SiO_2_ shell. Crucially, BT-BMT@SiO_2_ exhibits a symmetric Si-O-Si stretching peak at 800 cm^−1^, whereas this peak is absent in the uncoated sample, directly confirming the success of the SiO_2_ coating. The absorption peaks at 1260, 1097, and 1024 cm^−1^ may originate from residual carbonate ions or adsorbed oxygen-containing groups. Notably, the characteristic peaks attributed to BT-BMT lattice vibrations (635 cm^−1^ and 430 cm^−1^) retained their positions after coating, though their intensities decreased slightly, indicating that the SiO_2_ shell did not damage the intrinsic structure but merely produced dilution and screening effects. XRD results (Figure 1f) show that the diffraction peaks of BT-BMT are in good agreement with the perovskite ABO_3_ structure and are free of impurity phases [36]. No additional diffraction peaks were observed for BT-BMT@SiO_2_, confirming that the SiO_2_ is amorphous and that the coating process did not affect the crystal structure of BT-BMT, which is consistent with the conclusions from TEM.

The uniformity of inorganic filler dispersion directly affects the breakdown and energy storage performance of composite electrolytes. Figure 1g–i shows the cross-sectional SEM morphologies of pure PI, 0.25 vol.% BT-BMT/PI, and 0.25 vol.% BT-BMT@SiO_2_/PI. The pure PI film is dense and defect-free. At low loading levels, neither composite film exhibits particle agglomeration or significant voids. However, the BT-BMT@SiO_2_/PI system is more uniform and dense, reflecting the SiO_2_ shell’s role in improving interfacial compatibility, which is beneficial for maintaining high breakdown field strength and energy storage density. As the filler content increases (Figure S1), the differences between the two systems become more pronounced. In the BT-BMT/PI films (Figure S1a–c), the number of pits increased and their size expanded, severe particle agglomeration and numerous defects appeared at 1 vol.%. In contrast, the BT-BMT@SiO_2_/PI films (Figure S1d–f) consistently maintained good continuity, with no significant agglomeration even when the filler content reached 1 vol.%. The results indicate that the SiO_2_ coating effectively enhances the filler–matrix interfacial bonding, improving the structural uniformity and insulation reliability of the composite films.

**Figure 2:**
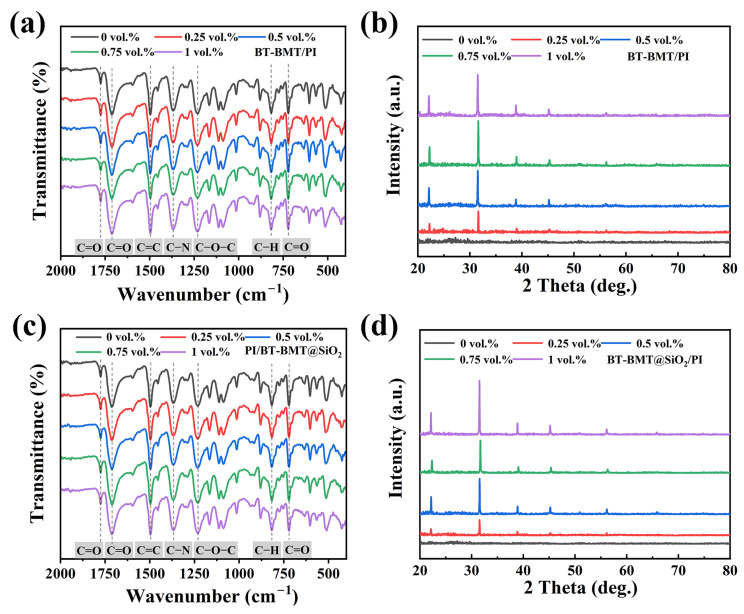
FT-IR spectra of the (**a**) BT-BMT/PI and (**c**) BT-BMT@SiO_2_/PI composites, XRD patterns of the (**b**) BT-BMT/PI and (**d**) BT-BMT@SiO_2_/PI composites.

The FT-IR spectra of pure PI, BT-BMT/PI, and BT-BMT@SiO_2_/PI composites are shown in Figure 2a,c. The absorption peaks at 1775 cm^−1^ and 1715 cm^−1^ correspond to the asymmetric and symmetric stretching vibrations of the C=O bond in the imide ring, respectively [50]. The absorption peaks near 1500 cm^−1^ and 815 cm^−1^ correspond to the C=C skeletal stretching vibration and the C-H out-of-plane bending vibration of the aromatic ring in PI. The absorption peaks near 1375 cm^−1^ and 1240 cm^−1^ originate from the C-N stretching vibration in the imide ring and the stretching vibration of the aromatic ether bond (C-O-C). The absorption peak near 720 cm^−1^ can be attributed to the out-of-plane bending vibration of the C=O group in the imide ring. The presence of these characteristic peaks confirms the successful synthesis of polyimide and its nanocomposites. A comparison of the FT-IR spectra of the samples before and after compositing reveals no significant shift in the positions of the characteristic absorption peaks, indicating that the introduction of BT-BMT and BT-BMT@SiO_2_ did not alter the chemical structure of PI. This provides a structural basis for the composite film to retain its excellent thermal and mechanical properties.

Figure 2b,d show the XRD patterns of the three types of composites mentioned above. The XRD pattern of pure PI exhibits only typical amorphous diffuse peaks, indicating a lack of long-range ordered arrangement in its molecular chains [50]. Following the introduction of BT-BMT and BT-BMT@SiO_2_ relaxor ferroelectric ceramic particles, a series of sharp diffraction peaks appeared in the XRD patterns of the composite dielectric. These peak positions fully matched the standard diffraction peaks of BT-BMT, and their intensities increased significantly with the increase in filler volume fraction. Furthermore, no diffraction signals attributable to SiO_2_ or other impurities were detected in the entire XRD pattern, confirming that the SiO_2_ coating is amorphous and maintains good structural stability during the composite preparation process.

**Figure 3:**
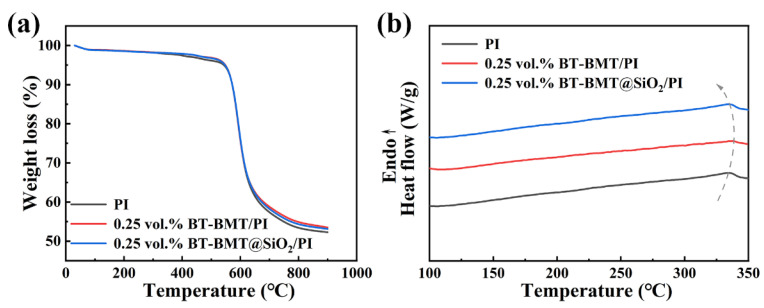
(**a**) TGA and (**b**) DSC curves of pure PI, 0.25 vol.% BT-BMT/PI, and 0.25 vol.% BT-BMT@SiO_2_/PI composites.

To evaluate the thermal stability of the composites, Figure 3 shows the thermogravimetric analysis (TGA) and differential scanning calorimetry (DSC) curves for three typical composites: pure PI, 0.25 vol.% BT-BMT/PI, and 0.25 vol.% BT-BMT@SiO_2_/PI. As shown in Figure 3a, the weight loss curves of the three samples are almost identical. When the temperature rises to approximately 570 °C, a distinct weight loss step appears in the curves, corresponding to the thermal decomposition of the PI matrix. This indicates that the introduction of small amounts of BT-BMT or BT-BMT@SiO_2_ fillers does not compromise the intrinsic thermal stability of PI. As shown by the DSC curves in Figure 3b, the glass transition temperature (*T*_g_) of pure PI is approximately 335 °C, while the *T*_g_ values of the two composites are 338 °C and 333 °C, respectively, with only a slight variation. This further demonstrates that the composite dielectric can maintain good thermal stability even at low filler contents.

**Figure 4:**
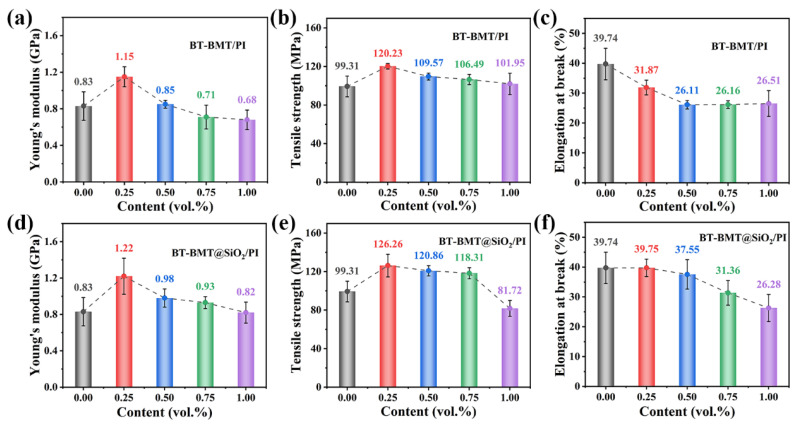
Variation in (**a**) Young’s modulus, (**b**) tensile strength, and (**c**) elongation at break of BT-BMT/PI composites as a function of filler content; variation in (**d**) Young’s modulus, (**e**) tensile strength, and (**f**) elongation at break of BT-BMT@SiO_2_/PI composites as a function of filler content.

There is a significant synergy between the mechanical properties and breakdown properties of composite dielectrics. Figure S2 shows typical stress–strain curves, and the Young’s modulus, tensile strength, and elongation at break calculated from these curves are shown in Figure 4. Figure 4a–c present the variation in these mechanical parameters in BT-BMT/PI composites as a function of filler content. As the BT-BMT content increases, the Young’s modulus and tensile strength of the composite exhibit an initial increase followed by a decrease, while the elongation at break continues to decrease. For pure PI, the Young’s modulus, tensile strength, and elongation at break are 0.83 GPa, 99.31 MPa, and 39.74%, respectively; when the filler content was 0.25 vol.%, these values were 1.15 GPa, 120.23 MPa, and 31.87%, respectively, with significant improvements in modulus and strength, and a slight decrease in elongation at break. This indicates that at low filler contents, BT-BMT particles can be uniformly dispersed in the PI matrix and form good interfacial bonding, thereby enhancing stiffness and strength while retaining good toughness. As the filler content continues to increase, the mechanical properties gradually deteriorate, which is mainly attributed to interfacial defects and stress concentration caused by particle agglomeration at high filler contents. SEM results also confirm this phenomenon.

Figure 4d–f correspond to the BT-BMT@SiO_2_/PI composite. Its Young’s modulus and tensile strength also exhibit an initial increase followed by a decrease, and the elongation at break gradually decreases. However, compared to the BT-BMT/PI system, the SiO_2_-coated core–shell particles further improved the mechanical properties of the composite system. This is attributed to the SiO_2_ shell effectively enhancing the dispersion of the filler and the interfacial bonding between the filler and the PI matrix. Taking 0.25 vol.% as an example, the Young’s modulus, tensile strength, and elongation at break of this composite reached 1.22 GPa, 126.26 MPa, and 39.75%, respectively, all of which were superior to those of the uncoated system. Even when the filler content was increased to 0.75 vol.%, the Young’s modulus and tensile strength remained at 0.93 GPa and 118.31 MPa, respectively, while the elongation at break remained above 30%, demonstrating good retention of mechanical properties.

**Figure 5:**
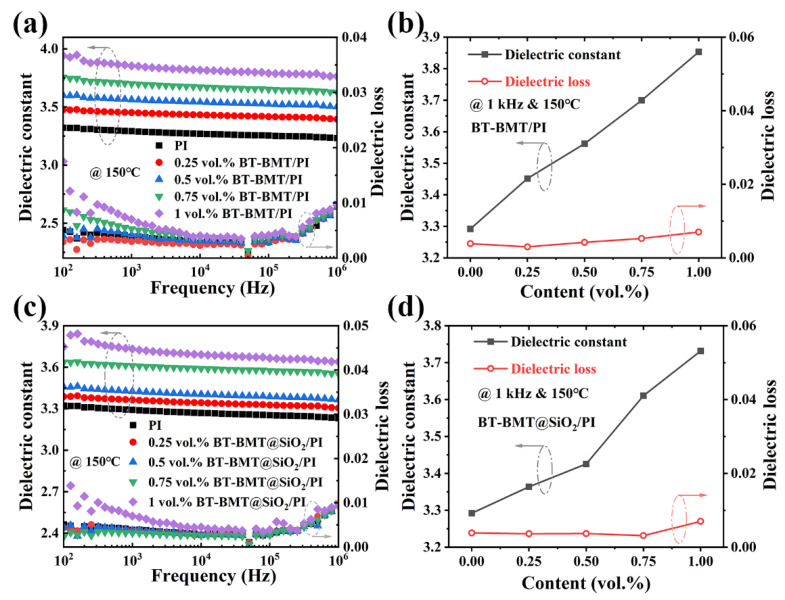
Variation in the dielectric constant and loss of BT-BMT/PI composites (**a**) with frequency and (**b**) with filler content at 1 kHz; and variation in the dielectric constant and loss of BT-BMT@SiO_2_/PI composites (**c**) with frequency and (**d**) with filler content at 1 kHz.

The variations in the dielectric constant and dielectric loss of the composite dielectric at 25 °C and 150 °C as a function of frequency and filler content are shown in Figure S3 and Figure 5. Overall, due to the suppressing effect of temperature on the dipole polarization response, the dielectric constant of the composite at 25 °C is slightly higher than that at 150 °C. For the BT-BMT/PI system (Figures S3a,b and Figure 5a,b), the dielectric constant of pure PI at 25 °C is 4.04, which increases to 4.19 and 4.42 upon the addition of 0.25 vol.% and 1 vol.% BT-BMT, respectively. At 150 °C, it increased from 3.32 for pure PI to 3.46 and 3.93, respectively. The increase in dielectric constant is primarily attributed to the high dielectric constant of the BT-BMT filler itself and the interfacial polarization effect between the filler and the PI matrix. Dielectric loss rose slightly with increasing filler content but remained below 0.02 at both room temperature and high temperatures, indicating that this composite system still exhibits excellent low-loss properties.

For the BT-BMT@SiO_2_/PI system (Figures S3c,d and Figure 5c,d), the lower dielectric constant of the SiO_2_ shell slightly attenuates the enhancement in dielectric constant. At a filler content of 0.25 vol.%, the dielectric constants at 25 °C and 150 °C increased to 4.10 and 3.39, respectively. At 0.5 vol.%, they further increased to 4.18 and 3.45. When the content reached 1 vol.%, they reached 4.34 and 3.84, respectively, representing increases of 0.30 and 0.52 compared to pure PI at room temperature and high temperature, respectively. This indicates that the SiO_2_ coating did not significantly weaken the dielectric constant enhancement effect of the composite system. Furthermore, the dielectric loss of the BT-BMT@SiO_2_/PI composite remained at a low level across the entire frequency range, and the difference in loss between 25 °C and 150 °C was significantly reduced compared to the uncoated system, demonstrating excellent thermal stability.

**Figure 6:**
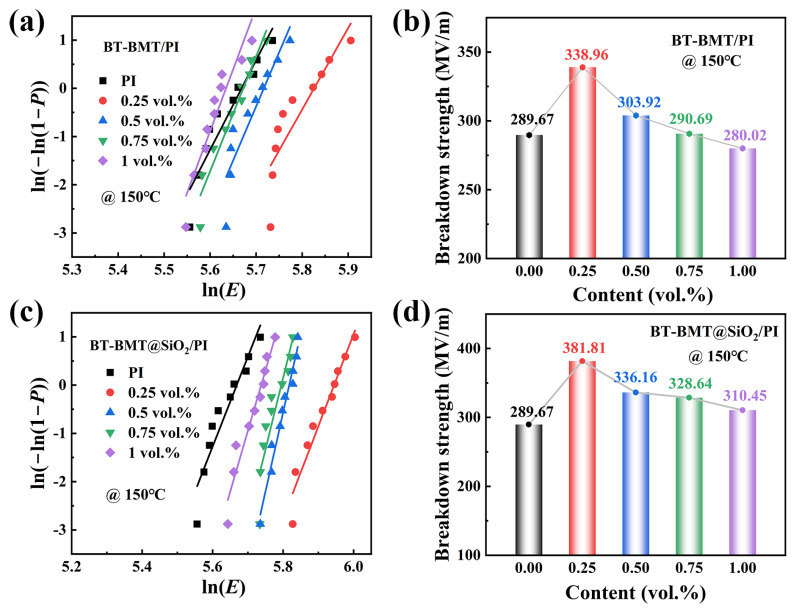
Variation in (**a**) the Weibull breakdown electric field distribution and (**b**) the breakdown field strength of BT-BMT/PI composites as a function of filler content at 150 °C; variation in (**c**) the Weibull breakdown electric field distribution and (**d**) the breakdown field strength of BT-BMT@SiO_2_/PI composites as a function of filler content at 150 °C.

Figures S4 and Figure 6 show the Weibull breakdown distributions and the variation in breakdown field strength with filler content for the composite dielectric at 25 °C and 150 °C, respectively. For the BT-BMT/PI system, the breakdown field strength first increases and then decreases with increasing filler content. With an optimal filler content of 0.25 vol.%, the composite exhibits breakdown strengths of 440.97 MV/m at 25 °C and 338.96 MV/m at 150 °C, which correspond to increases of 11.2% and 17% compared to those of neat PI (396.3 MV/m and 289.67 MV/m, respectively). When the content is increased to 0.5 vol.% and 0.75 vol.%, the breakdown field strength decreases slightly but remains higher than that of pure PI. At 1 vol.%, however, it drops to 375.24 MV/m (25 °C) and 280.02 MV/m (150 °C), which is lower than that of pure PI. Appropriate amounts of uniformly dispersed fillers can enhance the Young’s modulus and improve breakdown performance. Excessive amounts, however, lead to agglomeration, which exacerbates conductive pathways, defects, and interfacial polarization, thereby reducing dielectric strength.

The breakdown field strength of the BT-BMT@SiO_2_/PI system also exhibits an initial increase followed by a decrease, but the enhancement is more significant, and it remains higher than that of pure PI at all loading levels. With an optimal loading of 0.25 vol.%, the composite achieves breakdown strengths of 486.4 MV/m at 25 °C and 381.81 MV/m at 150 °C, corresponding to increases of 23% and 32% compared to neat PI. The SiO_2_ shell effectively suppresses charge injection and electric field concentration through dielectric buffering, surface defect repair, and interface optimization. Coupled with a significant increase in Young’s modulus, these effects collectively contribute to enhanced breakdown strength. Even at a high loading of 1 vol.%, the breakdown field strength still reaches 416.22 MV/m (25 °C) and 310.45 MV/m (150 °C), representing a 5% and 7% improvement over pure PI, respectively, confirming the effectiveness of the SiO_2_ coating strategy across a wide range of loading levels.

**Figure 7:**
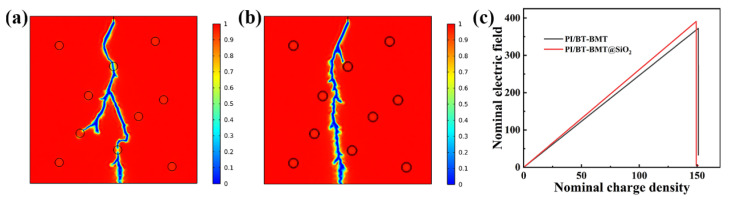
Phase-field simulations of breakdown in (**a**) 0.25 vol.% BT-BMT/PI and (**b**) 0.25 vol.% BT-BMT@SiO_2_/PI composites, and (**c**) the curve showing the variation in the nominal breakdown field strength with the nominal charge density.

To further verify the effectiveness of nanoparticle coating on breakdown performance, phase-field simulations were performed on BT-BMT/PI and BT-BMT@SiO_2_/PI composites. Specific parameters are provided in the Supporting Information [51,52]. Figure 7a,b show the simulated breakdown paths for the two systems, while Figure 7c displays the corresponding nominal electric field-charge density curves, where the peak of the nominal electric field represents the nominal breakdown field strength *E*_b_. The simulation results indicate that the BT-BMT@SiO_2_/PI composite exhibits a higher nominal *E*_b_ value, which is consistent with the experimental data.

Dielectric matching analysis indicates that while high-dielectric BT-BMT fillers can effectively increase the dielectric constant of the composite, they also tend to induce localized electric field concentration. Breakdown typically initiates at the site of the most severe electric field distortion. The strong localized electric fields generated by high-content BT-BMT particles induce the propagation of electric trees, thereby reducing the breakdown strength. In contrast, the SiO_2_ buffer layer can attenuate electric field distortions and alleviate localized electric field concentration, thereby maintaining or even enhancing the breakdown field strength. Furthermore, a smaller initial slope corresponds to a higher relative permittivity *ε*_r_ [52]. The BT-BMT/PI curve in Figure 7c exhibits a smaller initial slope, indicating a higher *ε*_r_, which is consistent with the dielectric test results.

Figures S5–S8 show the hysteresis loops of the composite dielectric at 150 °C. Compared with pure PI, the addition of BT-BMT and BT-BMT@SiO_2_ both increased the electric displacement, and the magnitude of this increase grew with increasing filler content, consistent with the trend in dielectric properties. The enhancement effect of BT-BMT was more pronounced, but the low breakdown field strength limited further increases in electric displacement.

**Figure 8:**
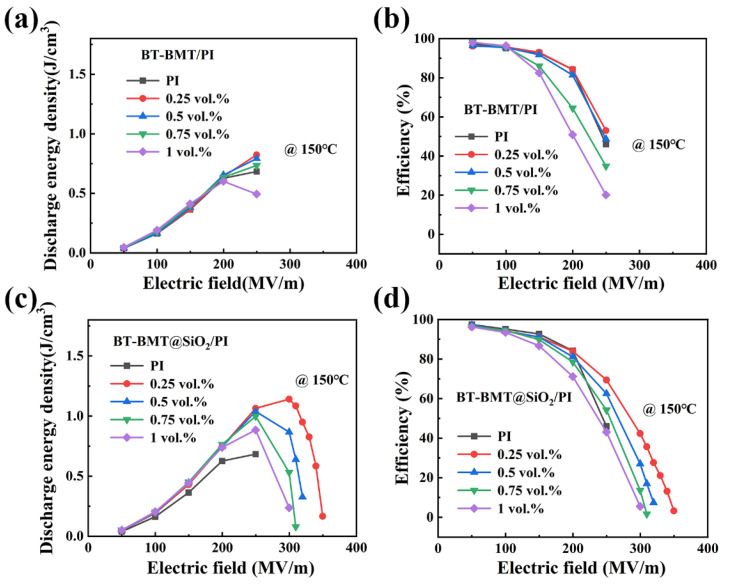
Variation in (**a**) discharge energy density and (**b**) efficiency of the BT-BMT/PI composite as a function of electric field strength at 150 °C; variation in (**c**) discharge energy density and (**d**) efficiency of the BT-BMT@SiO_2_/PI composite as a function of electric field strength at 150 °C.

Figure 8 shows the variation in energy storage density and efficiency with electric field, obtained by integrating the hysteresis loops. For both composite systems, the energy storage density and efficiency first increase and then decrease with increasing filler content. In the BT-BMT/PI system, the best performance was observed at 0.25 vol.%: at 150 °C and 250 MV/m, the energy storage density increased from 0.68 J/cm^3^ for pure PI to 0.82 J/cm^3^. At 0.75 vol.%, it decreased to 0.73 J/cm^3^, which was still higher than that of pure PI. At 1 vol.%, it deteriorated sharply to 0.6 J/cm^3^, with a significant drop in efficiency, attributed to increased defects and losses caused by agglomeration.

In comparison, the BT-BMT@SiO_2_/PI system demonstrated superior performance, with a higher breakdown field strength and a slower rate of efficiency decay. At 0.25 vol.%, the energy storage density reached 1.14 J/cm^3^, a 67% increase over pure PI. Even at a high loading of 1 vol.%, the energy storage density remained at 0.88 J/cm^3^, representing a 29% increase. The SiO_2_ buffer layer not only enhances the dielectric constant and breakdown field strength but also improves interfacial compatibility and dielectric matching, suppressing polarization loss, thereby achieving comprehensive optimization of energy storage performance.

**Figure 9:**
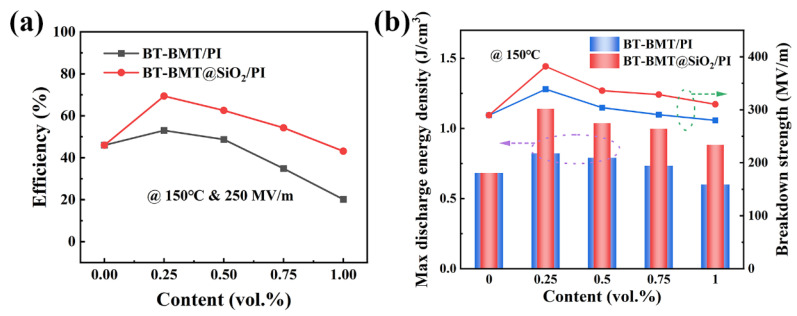
(**a**) Comparison of the efficiency of BT-BMT/PI and BT-BMT@SiO_2_/PI composites at 150 °C and 250 MV/m; (**b**) Comparison of the maximum discharge energy density and breakdown electric field strength of the above two composites at 150 °C as a function of filler content.

Figure 9 further compares the performance differences between the composite systems before and after coating, including the charge–discharge efficiency at 150 °C and 250 MV/m (Figure 9a), as well as the variation in maximum discharge energy density and breakdown electric field strength as a function of filler content at 150 °C (Figure 9b).

As shown in Figure 9a, the charge–discharge efficiency of both systems first increases and then decreases with filler content. However, BT-BMT@SiO_2_/PI outperforms BT-BMT/PI at all filler contents, with both systems achieving their optimal efficiencies at 0.25 vol.%, at 69.4% and 52.9%, respectively. The improvement in the efficiency of the coated system stems from the SiO_2_ shell’s enhancement of interfacial compatibility and dielectric matching.

The trends in discharge energy density and breakdown electric field strength align with those of efficiency (Figure 9b), with BT-BMT@SiO_2_/PI exhibiting significantly superior performance to BT-BMT/PI. At 0.25 vol.%, the former exhibited a breakdown electric field strength and maximum discharge energy density of 381.81 MV/m and 1.14 J/cm^3^, respectively, representing increases of approximately 12% and 39% compared to the latter’s 338.96 MV/m and 0.82 J/cm^3^. This indicates that the SiO_2_ coating strategy can effectively balance breakdown enhancement and increased energy storage density at low filler content.

## 4. Conclusions

In this work, two high-temperature-resistant composite dielectrics, BT-BMT/PI and core–shell BT-BMT@SiO_2_/PI, were fabricated by incorporating relaxor ferroelectric nanoparticles into a polyimide (PI) matrix. Both composites showed enhanced dielectric constants relative to pure PI. Compared with the uncoated system, the ~4 nm SiO_2_ shell in BT-BMT@SiO_2_/PI significantly improved filler–matrix interfacial compatibility, preserved the uniform and dense film structure, and increased the Young’s modulus. This dielectric buffer layer also effectively mitigated the permittivity mismatch between the high-*ε*_r_ BT-BMT core and the PI matrix, suppressing local electric field concentration and thereby raising the breakdown strength. Consequently, the 0.25 vol.% BT-BMT@SiO_2_/PI composite exhibits a discharge energy density of 1.14 J/cm^3^ (a 67% increase over pure PI) at 150 °C and 250 MV/m, which is superior to the BT-BMT/PI system. These results demonstrate that the synergistic design of a relaxor ferroelectric ceramic core with an inorganic SiO_2_ shell provides an effective strategy for developing high-temperature, high-energy-density polymer-based composite dielectrics.

## Data Availability

The original contributions presented in this study are included in the article/Supplementary Materials. Further inquiries can be directed to the corresponding authors.

## Supplementary Materials

The following supporting information can be downloaded at: https://www.mdpi.com/article/10.3390/polym18141784/s1. Refs. [53,54] are cited in the Supplementary Materials.
