# Supplementary material for: High-Temperature Energy Storage Performance of Polyimide Nanocomposites Enhanced by Core–Shell BT-BMT@SiO2
Source: Polymers (Basel). 2026 Jul 21;18(14):1784. doi: 10.3390/polym18141784 (PMC13418836; doi:10.3390/polym18141784)
Supplement: Supplementary file 1 [file polymers-18-01784-s001.zip › polymers-4431569-supplementary.pdf]

Supporting Information

High-Temperature Energy Storage Performance of Polyimide Nanocomposites Enhanced by Core–Shell BT-BMT@SiO_2

Zunpeng Feng [1,2], Xingyu Hou [1], Sitian Ren [1], Xinyao Zhuang [1], Wei Chen [1], Haoran Liu [1], Chaoqiong Zhu [1,2], Ziming Cai [1,2,] and Peizhong Feng [1,2,*]*

1 School of Materials Science and Physics, China University of Mining and Technology, Xuzhou 221116, China; fengzunpeng@cumt.edu.cn (Z.F.); hxy_980522@163.com (X.H.); ren041346@163.com (S.R.); 14240159@cumt.edu.cn (X.Z.); 18895795431@163.com (W.C.); ts25180114p31@cumt.edu.cn (H.L.); zhucq@cumt.edu.cn (C.Z.)

2 Jiangsu Key Laboratory for Clean Utilization of Carbon Resources, Xuzhou 221116, China

* Correspondences: zmcai@cumt.edu.cn (Z.C.); pzfeng@cumt.edu.cn (P.F.)

A Phase-Field Model for the Formation and Propagation of Electrical Dendrites in Dielectric Materials

Similar to phase-field models of mechanical fracture,[1, 2] a time- and space-dependent scalar field $s(x, t)$ is introduced to characterize the formation and propagation of electrical branches in the dielectric. The value of s varies continuously between 1 and 0. s=1 means intact, while s=0 means breakdown. The completely damaged region

in the dielectric becomes a conductor. In numerical simulations, the dielectric constant of this region is assigned a very large but finite value of ε_0/η, where ε_0 is the initial dielectric constant and η is a sufficiently small number, taken as 10^{-4} in this model. Thus, for other material states, the dielectric constant can be interpolated using the following equation:

$$\varepsilon(s) = \frac{\varepsilon_0}{f(s) + \eta} \qquad (1)$$

Here, $f(s) = 4s^3 - 3s^4$. Electrical branching occurs when this process leads to a decrease in the total free energy of the dielectric material, which can be expressed as:

$$\Pi[s, \phi] = \int_{\Omega} [W_{\text{es}}(E, s) + W_{\text{d}}(s) + W_{\text{i}}(\nabla s)]\, \mathrm{d}V \qquad (2)$$

In particular, $W_{\text{es}}(E, s) = -\frac{\varepsilon}{2} E \cdot E$ is the electrostatic energy per unit volume; $W_{\text{d}}(s) = W_{\text{c}}[1 - f(s)]$ is the breakdown energy function, where W_{c} represents the critical electrostatic energy density; and $W_{\text{i}}(\nabla s) = \frac{\Gamma}{4} \nabla s \cdot \nabla s$ is the gradient energy at the phase interface. It should be emphasized that the material parameter Γ is approximately equal to the breakdown energy. According to linear kinetic theory: $\partial s/\partial t = -m\, \delta\Pi/\delta s$, the breakdown variable can be expressed as:

$$\frac{1}{m}\frac{\partial s}{\partial t} = \frac{\varepsilon'(s)}{2} \nabla\phi \cdot \nabla\phi + W_{\text{c}} f'(s) + \frac{\Gamma}{2} \nabla^2 s \qquad (3)$$

In particular, the damage rate m is a parameter used to characterize the propagation rate of electrical branches. To simplify calculations, all quantities in the model are normalized as follows: length is normalized by the characteristic width l of the damaged region ($l = \sqrt{\Gamma/W_{\text{c}}}$), energy density by W_{c}, time by $1/mW_{\text{c}}$, and potential

by $\sqrt{\Gamma/\varepsilon_0}$. Finally, the normalized governing equation for electrical branch propagation can be written as:

$$\bar{\nabla}\cdot\left[\frac{1}{f(s)+\eta}\bar{\nabla}\bar{\phi}\right]=0 \qquad (4)$$

$$\frac{\partial s}{\partial \bar{t}}=-\frac{f'(s)}{2[f(s)+\eta]^2}\bar{\nabla}\bar{\phi}\cdot\bar{\nabla}\bar{\phi}+f'(s)+\frac{1}{2}\bar{\nabla}^2 s \qquad (5)$$

The normalized variables are indicated by a horizontal line. By setting appropriate boundary conditions and initial values, equations (4) and (5) above can be used to solve for the unknown dimensionless field variables $\bar{\phi}(\bar{x},\bar{t})$ and $s(\bar{x},\bar{t})$. Since this model focuses primarily on investigating the mechanisms of electric tree formation and propagation in dielectrics, and to conserve computational resources, all simulations are conducted in a two-dimensional domain. The phase-field simulation is performed using the finite element software COMSOL Multiphysics. To characterize defects in the dielectric and introduce randomness into the simulation, a small random perturbation is added to the second term on the right-hand side of Equation (5).

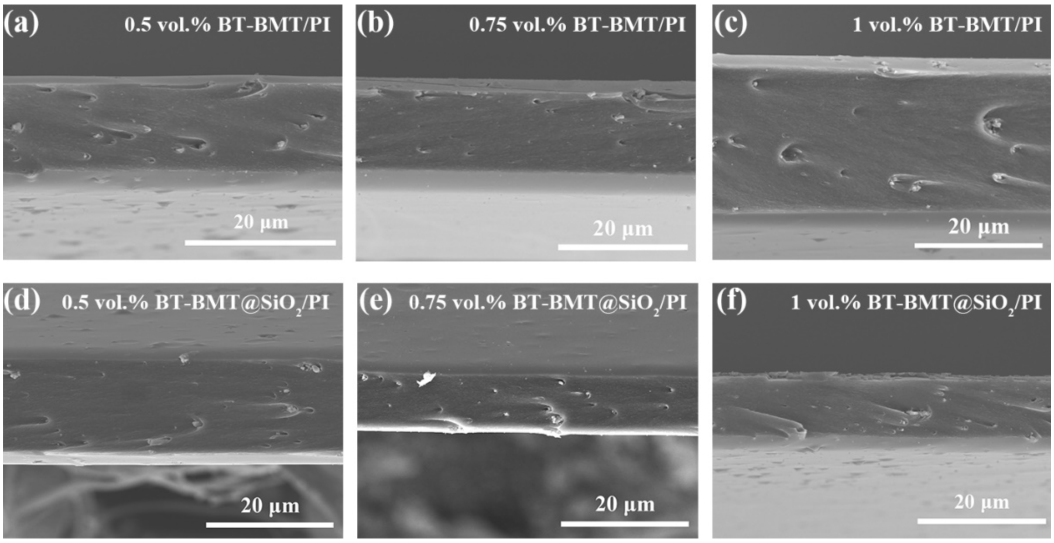

Figure S1. (a) Cross-sectional SEM images of BT-BMT/PI composite dielectrics at 0.5 vol.%, (b) 0.75 vol.%, and (c) 1 vol.% BT-BMT/PI composite dielectric cross-sectional SEM images; (d) 0.5 vol.%, (e) 0.75 vol.%, and (f) 1 vol.% BT-BMT@SiO_2/PI composite dielectric cross-sectional SEM images.

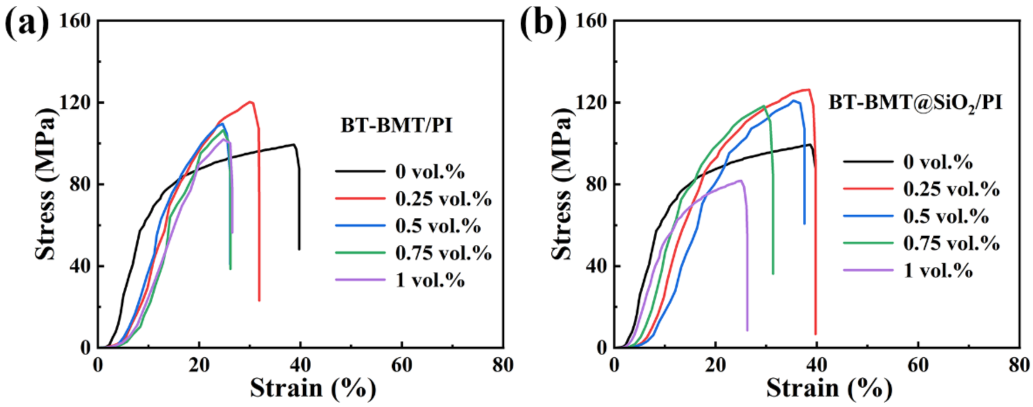

Figure S2. Stress-strain curves for (a) BT-BMT/PI and (b) BT-BMT@SiO_2/PI composite dielectrics.

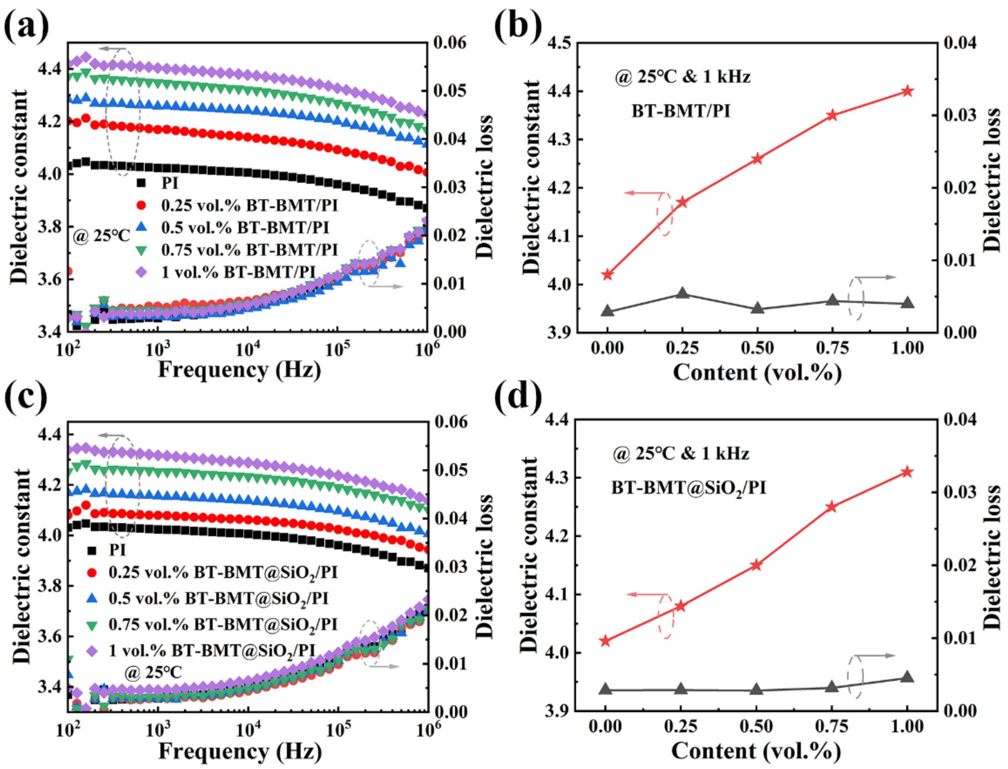

Figure S3. (a) Frequency dependence of the dielectric constant and loss of the BT-BMT/PI composite dielectric at 25°C, and (b) dependence of the dielectric constant and loss on filler content at 1 kHz; (c) Frequency dependence of the dielectric constant and loss of the BT-BMT@SiO_2/PI composite dielectric at 25°C, and (d) the variation of the permittivity and loss at 1 kHz as a function of filler content.

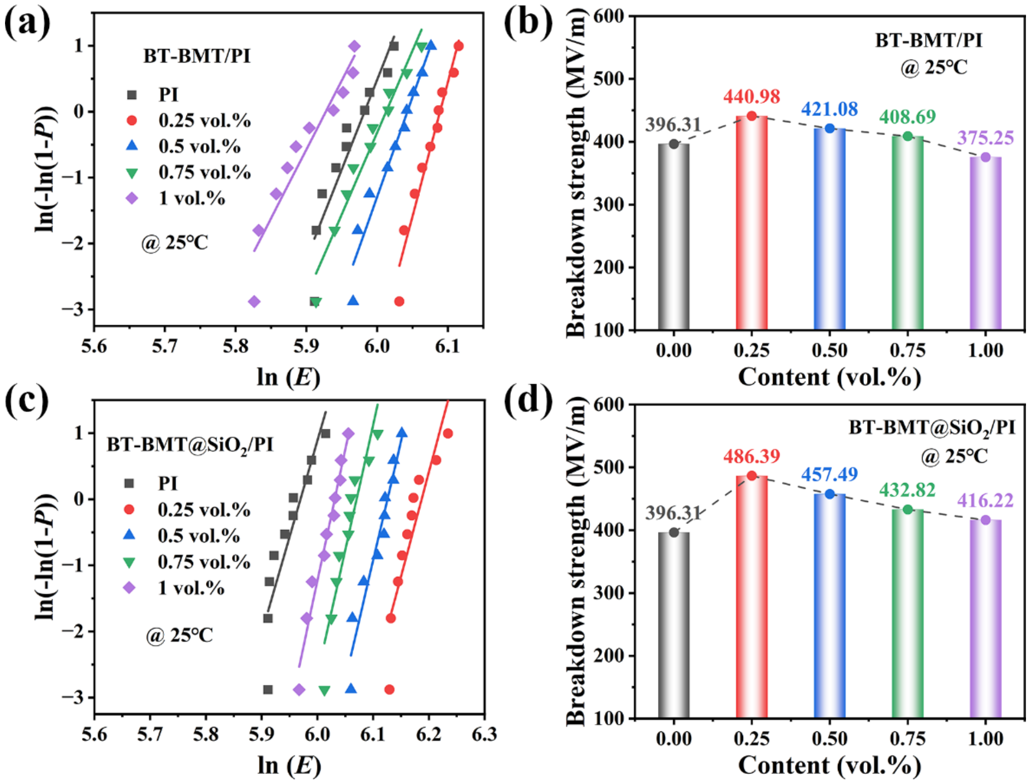

Figure S4. (a) Weibull breakdown field strength of the BT-BMT/PI composite dielectric at 25°C, and (b) variation of the breakdown field strength with filler content; (c) Weibull breakdown field strength of the BT-BMT@SiO_2/PI composite dielectric at 25°C, and (d) variation of the breakdown field strength with filler content.

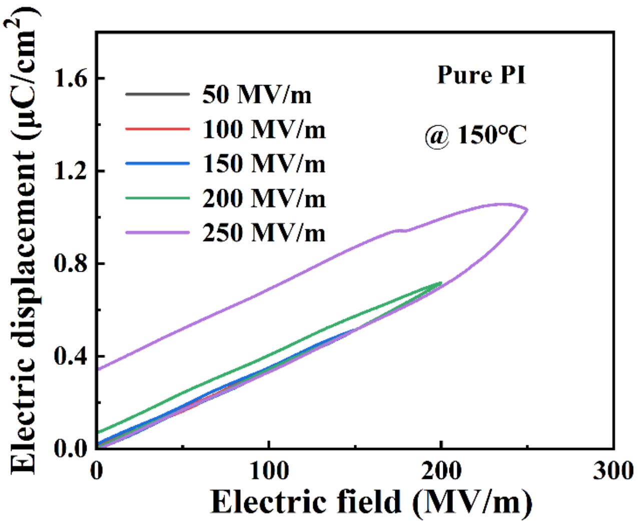

Figure S5. Hysteresis loops of pure PI at 150℃ under different electric field strengths.

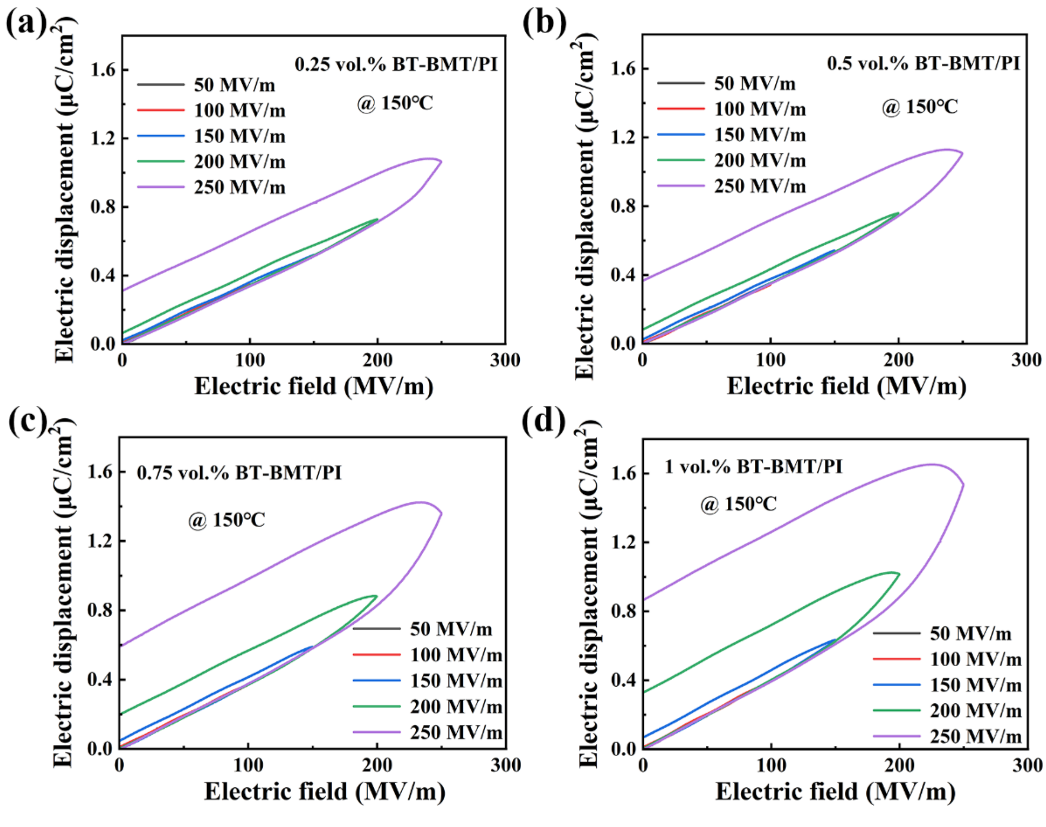

Figure S6. Hysteresis loops of (a) 0.25 vol.%, (b) 0.5 vol.%, (c) 0.75 vol.%, and (d) 1 vol.% BT-BMT/PI composite dielectrics at 150℃ under different electric field strengths.

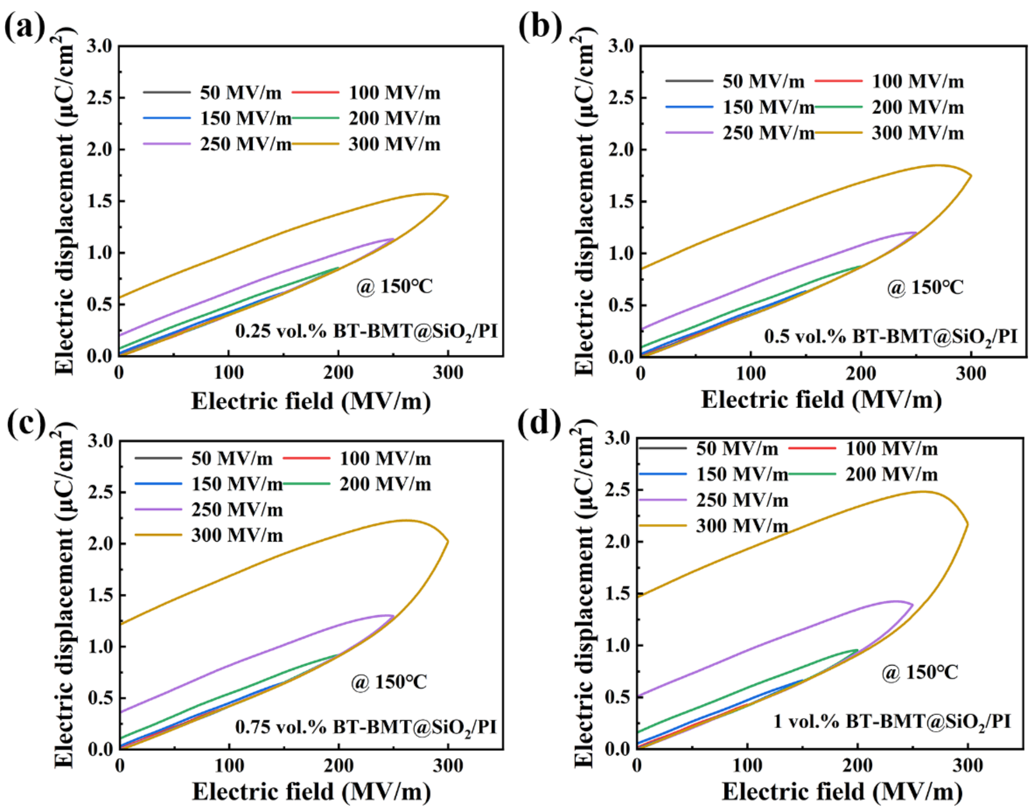

Figure S7. Hysteresis loops of (a) 0.25 vol.%, (b) 0.5 vol.%, (c) 0.75 vol.%, and (d) 1 vol.% BT-BMT@SiO_2/PI composite dielectrics at 150℃ under different electric field strengths.

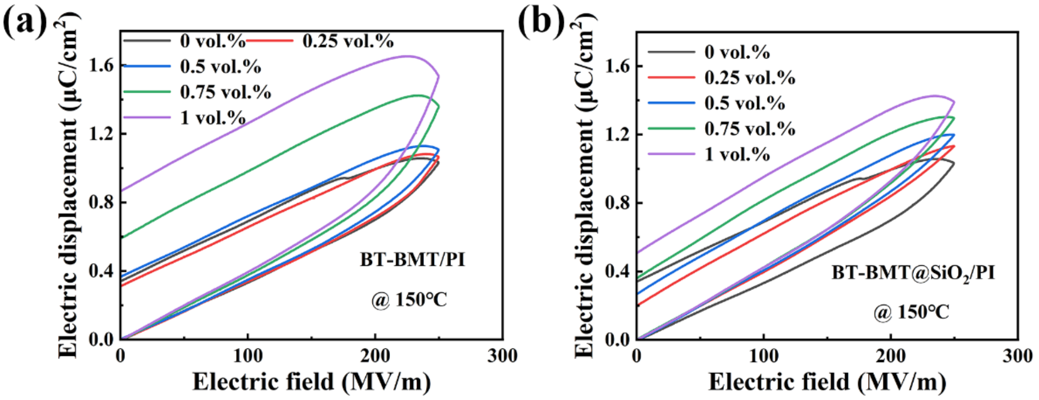

Figure S8. Hysteresis loops of (a) BT-BMT/PI and (b) BT-BMT@SiO_2/PI composite dielectrics at 150℃ and 250 MV/m.

References

(1) Cai, Z.; Wang, X.; Luo, B.; Hong, W.; Wu, L.; Li, L. Dielectric response and breakdown behavior of polymer-ceramic nanocomposites: The effect of nanoparticle distribution. *Compos. Sci. Technol.* **2017**, *145*, 105–113.

(2) Cai, Z.; Wang, X.; Luo, B.; Hong, W.; Wu, L.; Li, L. Nanocomposites with enhanced dielectric permittivity and breakdown strength by microstructure design of nanofillers. *Compos. Sci. Technol.* **2017**, *151*, 109–114.
